# Immune modulation in transplant medicine: a comprehensive review of cell therapy applications and future directions

**DOI:** 10.3389/fimmu.2024.1372862

**Published:** 2024-04-08

**Authors:** Leonard Knoedler, Jillian Dean, Fortunay Diatta, Noelle Thompson, Samuel Knoedler, Richmond Rhys, Khalil Sherwani, Tobias Ettl, Simon Mayer, Florian Falkner, Katja Kilian, Adriana C. Panayi, Jasper Iske, Ali-Farid Safi, Stefan G. Tullius, Siba Haykal, Bohdan Pomahac, Martin Kauke-Navarro

**Affiliations:** ^1^Department of Plastic, Hand and Reconstructive Surgery, University Hospital Regensburg, Regensburg, Germany; ^2^Division of Plastic Surgery, Department of Surgery, Yale New Haven Hospital, Yale School of Medicine, New Haven, CT, United States; ^3^School of Medicine, University of Pittsburgh, Pittsburgh, PA, United States; ^4^University of Toledo College of Medicine and Life Sciences, Toledo, OH, United States; ^5^Department of Hand, Plastic and Reconstructive Surgery, Burn Center, Berufsgenossenschaft (BG) Trauma Center Ludwigshafen, University of Heidelberg, Ludwigshafen, Germany; ^6^Department of Dental, Oral and Maxillofacial Surgery, Regensburg, Germany; ^7^Department of Cardiothoracic and Vascular Surgery, Deutsches Herzzentrum der Charité, Berlin, Germany; ^8^Charité Universitätsmedizin Berlin, Berlin, Germany; ^9^Faculty of Medicine, University of Bern, Bern, Switzerland; ^10^Craniologicum, Center for Cranio-Maxillo-Facial Surgery, Bern, Switzerland; ^11^Division of Transplant Surgery, Department of Surgery, Brigham and Women’s Hospital, Harvard Medical School, Boston, MA, United States

**Keywords:** solid organ transplantation, SOT, vascularized composite allotransplantation, VCA, cellular therapies

## Abstract

Balancing the immune response after solid organ transplantation (SOT) and vascularized composite allotransplantation (VCA) remains an ongoing clinical challenge. While immunosuppressants can effectively reduce acute rejection rates following transplant surgery, some patients still experience recurrent acute rejection episodes, which in turn may progress to chronic rejection. Furthermore, these immunosuppressive regimens are associated with an increased risk of malignancies and metabolic disorders. Despite significant advancements in the field, these IS related side effects persist as clinical hurdles, emphasizing the need for innovative therapeutic strategies to improve transplant survival and longevity. Cellular therapy, a novel therapeutic approach, has emerged as a potential pathway to promote immune tolerance while minimizing systemic side-effects of standard IS regiments. Various cell types, including chimeric antigen receptor T cells (CAR-T), mesenchymal stromal cells (MSCs), regulatory myeloid cells (RMCs) and regulatory T cells (T_regs_), offer unique immunomodulatory properties that may help achieve improved outcomes in transplant patients. This review aims to elucidate the role of cellular therapies, particularly MSCs, T cells, T_regs_, RMCs, macrophages, and dendritic cells in SOT and VCA. We explore the immunological features of each cell type, their capacity for immune regulation, and the prospective advantages and obstacles linked to their application in transplant patients. An in-depth outline of the current state of the technology may help SOT and VCA providers refine their perioperative treatment strategies while laying the foundation for further trials that investigate cellular therapeutics in transplantation surgery.

## Introduction

Over the last thirty years, the field of plastic surgery has adopted principles from solid organ transplantation (SOTs), particularly as vascularized composite allotransplantation (VCA) has become more prominent (1–3). This biotechnology offers unique clinical benefits, especially in restoring form and functionality in patients with complex and devastating deformities that cannot be adequately reconstructed using conventional strategies such as local tissue rearrangement or free flap reconstruction (4). One of the major challenges of transplantation is rejection of the allograft and the need for life-long multidrug immunosuppression that often has significant systemic side effects.

Current standard immunosuppressive regimens include corticosteroids, tacrolimus, and mycophenolate mofetil (MMF), among other immunosuppressants (4–6). While modern immunosuppressive drugs have effectively reduced rejection rates, prolonged use has been implicated with increased risk of metabolic disorders, infections, and cancer (5). Tacrolimus has been linked with pancreatic beta cell toxicity (7), increasing the risk of post-transplant diabetes by 25% compared to cyclosporine-based regimens (8). Antithymocyte globulin (ATG) has also been shown to predispose post-transplant patients to opportunistic infections, such as cytomegalovirus (CMV) or fungal infections (9). In this context, a 2019 study enrolled 2,495 liver transplant patients receiving tacrolimus-based immunosuppression and revealed that 19.7% of study participants developed malignancies over a mean follow-up period of 5.6 years (10).

Recent advances suggest cellular therapy as a promising solution to address these ongoing challenges in transplantation surgery (11). This strategy involves administering viable cell subsets stemming from autologous, allogeneic, and xenogeneic sources (11–13). In contrast to conventional immunosuppressants that are non-specific modulators of the immune response (14), cellular therapies are intended to be more specific and targeted, thereby reducing systemic side effects (1). The main goal of these therapies is to establish a stable state of immune homeostasis, inhibiting both graft-versus-host and host-versus-graft reactions, while reducing the need for long-term conventional immunosuppressive treatments (15). This review is intended to provide a comprehensive overview of cellular therapies for the treatment of SOT and VCA patients, reviewing their key functions, clinical use cases, and potential applications. Ultimately, this line of research may unlock untapped therapeutic potential and help SOT and VCA providers optimize their perioperative treatment protocols.

## Perioperative standard pharmacological therapy in transplantation surgery

Options for induction therapy include ATGs, anti-IL2 receptor antibodies, and anti-CD3 antibodies (16), all of which have been shown to significantly reduce acute rejection rates in the short-term perioperative setting (17, 18). The 2021 Organ Procurement and Transplantation Network (OPTN) Report indicated that 67.5% of adult transplant patients were discharged on a triple therapy maintenance regimen consisting of tacrolimus, MMF, and corticosteroids, while the remaining 25.6% of patients were discharged on dual therapy (tacrolimus-MMF) (19). For VCAs, recent research has shed light on the primary targets of rejection reactions, identifying the mucosa (20) and skin (21) as key areas of concern. Induction therapy, akin to SOTs, is frequently based on ATG (22). Despite the adoption of SOT triple therapy as the standard maintenance regimen for the majority of VCA patients, the occurrence of acute rejection within the first-year post-transplant remains high, exceeding 80% (23). Furthermore, while chronic rejection rates in VCA patients have not been definitively established, approximately 85% of VCA recipients experience at least one episode of acute rejection during their first year of transplantation (24, 25).

## Cellular therapeutic strategies in transplantation patients

Over the past three decades, cellular therapies have emerged as a novel approach to address the persisting challenges linked to multidrug and lifelong immunosuppression (26, 27). The concept of cellular therapies is centered around the infusion of immunomodulating cells. Such cell populations target specific immunologic pathways linked with allograft rejection and off-target systemic side effects (28). In preclinical VCA models, T_regs_ (29), CAR-T cells (30, 31), MSCs (32, 33), and RMCs (34) have demonstrated to improve allograft acceptance and minimize systemic toxicity.

### Regulatory T cells (T_regs_) - general cellular characteristics

T_regs_ are a specialized subset of T cells that play a crucial role in modulating the immune response to promote anti-inflammatory outcomes, serving as one of the primary mediators of physiological immune suppression (35). T_regs_ are broadly classified into thymus-derived T_regs_ (nT_regs_), which develop in the thymus, and induced T_regs_ (iT_regs_), which differentiate from conventional T cells in the periphery under specific conditions, such as exposure to antigens in a tolerogenic context (36). Peripheral T_regs_ (pT_regs_) are a type of iT_reg_ that arises in the periphery following antigenic stimulation, and can be distinguished by specific markers like neuropilin-1 and Helios in conjunction with Foxp3 expression, the defining transcription factor for regulatory T cell lineage (36). Contrary to the previous classification based on CD45RA and Foxp3 expression, recent findings suggest that the expression of neuropilin-1 (Nrp-1) and Helios can more accurately distinguish nT_regs_ from pT_regs_ *in vivo*, with the former typically expressing higher levels of Nrp-1 (37).

T_regs_ are characterized by the transcription factor Foxp3, which is crucial for their suppressive function and the maintenance of immune homeostasis (38). pTregs are increasingly recognized for their role in peripheral tolerance (39), especially at mucosal sites and during certain inflammatory conditions (40). It is important to note that not all regulatory T cells express Foxp3 (41). Type 1 regulatory T (Tr1) cells, for instance, do not express Foxp3 but instead secrete high levels of IL-10 and contribute to immune tolerance primarily through IL-10 and TGF-β dependent pathways, contributing to their regulatory capabilities (42). Thus, the functional mechanisms of T_regs_ are more diverse than solely suppressive interactions, encompassing a range of anti-inflammatory pathways. These characteristics make Tr1 cells pivotal for the maintenance of long-term tolerance, particularly in SOT (43), however the production of antigen-specific Tr1 cells alone is inadequate, and it is also essential to reduce the activity of effector T cells (T_effs_) (44). The clinical significance of Tr1 cells is highlighted by emerging evidence and ongoing clinical trials that are exploring their potential in inducing operational tolerance. Tr1 cells’ ability to be induced and expanded *ex vivo* (45), along with their stable phenotype in inflammatory post-transplant environments (46), positions them as promising candidates for achieving and sustaining this state of tolerance.

### Tr1 cells in SOT patient populations

Tr1 cells have been associated with positive outcomes in transplantation, including stable graft function and tolerance (47). The induction of Tr1 cells can be achieved through several methods, often involving cytokines like IL-10 and IL-27 (48), signaling through specific surface molecules (47), and interactions with dendritic cells (DCs) (49). This versatility makes Tr1 cells a valuable target for clinical applications, particularly in transplantation where they can potentially prevent organ rejection and reduce the need for long-term immunosuppression.

In a study conducted by Jofra et al., Tr1 cell immunotherapy was employed to enhance transplant tolerance in a mouse model of pancreatic islet transplantation (50). They discovered that the LAG3^+^ cells from tolerant mice, which were enriched in Tr1 cells, expressed high levels of IL-10, with approximately 17.5 ± 2.2% FoxP3^+^ cells. This indicated a significant presence of Tr1 cells. The role of these Tr1 cells in maintaining transplant tolerance was further elucidated as blocking these cells with an anti-LAG3 mAb led to the loss of transplant tolerance, highlighting their crucial role. Moreover, when Tr1 cell therapy was administered to IL-10-deficient mice, the mice failed to develop transplant tolerance, suggesting that host IL-10 is essential for the induction of tolerance (50).

Another key finding was the *de novo* generation of Tr1 cells in the recipients. The study showed that transferred Tr1 cells did not home to the graft or its draining lymph nodes due to the lack of CD62L expression, which is necessary for adhesion to high endothelial venules. An increase in host LAG3^+^ CD4 T cells in Tr1-treated recipient mice indicated that the therapy led to an induction of Tr1 cells in the host. This was further supported by the increased number of IL-10^+^ cells expressing LAG-3 in the spleens of IL-10-reporter mice that received Tr1 cell therapy (50). The study also addressed concerns about the safety and stability of Tr1 cell therapy during acute viral infections. To test this, researchers infected Tr1-treated tolerant mice with lymphocytic choriomeningitis virus (LCMV) and observed that the tolerance induced by Tr1 cell therapy was not detrimental to the host’s ability to control the viral infection. The Tr1 treated mice successfully survived the infection and maintained the graft. Importantly, the tolerogenic state did not compromise the development of effective antiviral immune responses, as evidenced by the maintenance of both CD8 and CD4 anti-LCMV responses (50). This suggests that Tr1 cell therapy can establish a stable and safe transplant tolerance that does not inhibit the host’s ability to respond to acute viral infections.

A study of Tr1 cell therapy for type 1 diabetes in the context of islet transplantation presents compelling data across different experimental models (51). In the less stringent transplant scenario involving BALB/c mice receiving C57BL/6 islets, polyclonal Tr1 cells demonstrated notable efficacy. Specifically, after the transfer of these cells into diabetic BALB/c mice, 62.5% (5 out of 8) did not reject the graft within 25-days post-transplant. Furthermore, a long-term graft survival rate of 50% was observed at the 100-day mark. This contrasted sharply with the outcomes in a more stringent model, where C57BL/6 mice received BALB/c islets. In this setting, all recipient mice, regardless of receiving polyclonal Tr1 cells or Th0 cells, uniformly rejected the graft promptly, underscoring the limited effectiveness of polyclonal Tr1 cells in more challenging transplant environments (51).

A significant enhancement in outcomes was noted with the use of antigen (Ag)-specific Tr1 cells in the so-called stringent model (51). All mice receiving these Ag-specific Tr1 cells maintained functional grafts 25-days post-transplant, and 40% sustained long-term graft survival up to 100 days. The study also highlighted the critical role of IL-10 in sustaining transplant tolerance. This was particularly evident when all mice treated with an αIL-10 receptor monoclonal antibody experienced rapid graft rejection, confirming the IL-10-dependent nature of the tolerance induced by Tr1-cell therapy. An important aspect of the study was the emphasis on the donor antigen specificity of the Tr1 cells. When BALB-specific Tr1 cells were transferred into B6 mice receiving C3H islets, the grafts were rejected, illustrating that the therapeutic advantage of Tr1 cells is closely tied to their donor antigen specificity. Taken together, these findings suggest that Tr1-cell therapy, especially when tailored to the donor antigen, can effectively induce IL-10–dependent tolerance in challenging models of islet transplantation (51).

The findings from the study on Tr1 cell therapy in the context of type 1 diabetes have broader implications for SOTs. By producing high levels of IL-10 and suppressing immune responses against specific antigens, Tr1 cells could reduce the need for generalized immunosuppression (52). This approach is particularly valuable in SOTs where the risk of chronic rejection and drug-induced complications remains a significant challenge (53). The potential to tailor Tr1 cells to the specific antigens of the transplanted organ opens a pathway to more personalized and effective transplant therapies, enhancing graft survival while maintaining overall immune system integrity (54). Clinically, protocols have been developed for the *in vitro* expansion of Tr1 cells (55, 56), enabling their use as therapeutic products in immune-mediated diseases and transplantation (42). However, challenges remain, such as the need for specific biomarkers to isolate pure Tr1 cells and the limited expansion capacity of current protocols (47).

As of now, clinical trials have established the safety and effectiveness of Tr1 cell therapy in certain contexts. A single-center, non-randomized, and prospective phase I–II trial (ALT-TEN protocol, IS/11/6172/8309/8391) has been conducted, where patients with high-risk or advanced-stage hematologic malignancies received both haploidentical hematopoietic stem cell transplantation (haplo-HSCT) and Tr1-cell therapy (57). This combined treatment has shown potential in enhancing immune system reconstitution and reducing both the risk of disease relapse and the occurrence of GVHD following HSCT. Additionally, a new Phase I trial (NCT03198234) is underway, testing the use of T-allo10 cells to prevent GVHD after mismatched related or unrelated hematopoietic stem cell transplantation (HSCT) without any pre-treatment in cases of hematologic malignancies (58). Preliminary results from this trial have been encouraging and deemed safe, yet further analysis is necessary to fully assess the clinical outcomes.

The clinical trials investigating Tr1-cell therapy, particularly those in the context of HSCT (59), provide a foundational understanding that can be applied to SOT and VCA. In SOT, the goal of Tr1 cell therapy would be to induce specific tolerance to the transplanted organ, thereby minimizing the risk of organ rejection while potentially reducing the need for long-term systemic immunosuppressive drugs. The ability of Tr1 cells to regulate the immune response by producing anti-inflammatory cytokines, and their stability in the presence of immunosuppressive drugs, as observed in kidney transplant patients, are particularly promising for SOT (47). These properties may help to maintain the delicate balance necessary to prevent organ rejection while avoiding the complications of over-suppression, such as susceptibility to infections and malignancies. In VCA, the immunological challenges are even greater due to the diversity of tissue types involved, each with different antigenic profiles (10, 60). The immunomodulatory capabilities of Tr1 cells could be harnessed to promote graft tolerance and reduce the incidence of both acute and chronic rejection, which is vital for the long-term success of these grafts. The preliminary results of Tr1-cell therapy in preventing GvHD in HSCT patients indicates its potential for application in SOT and VCA, where similar mechanisms of immune-mediated rejection occur (61). By extending the application of Tr1-cell therapy to these areas, there is a potential to significantly improve patient outcomes through targeted immune regulation.

Looking ahead, the potential of genetically engineered Tr1 cells, such as those modified with CAR constructs (CAR-Tr1 cells), represents a promising avenue of research (62). CAR-Tr1 cells could offer a more targeted approach to immunomodulation in transplantation, possibly enhancing the specificity and efficacy of cellular therapies (47). Despite the progress made, further research is essential to fully understand Tr1 cells’ regulatory mechanisms and to harness their therapeutic potential reliably. Further, the induction of Tr1 cells could be a therapeutic lever that offers advantages over traditional FoxP3^+^ T_reg_ therapies (40). While FoxP3^+^ T_regs_ are essential for establishing tolerance (63), Tr1 cells have been identified as key players in sustaining this tolerance long-term (59). This understanding may reshape therapeutic strategies in SOT, with an emphasis on harnessing the unique properties of Tr1 cells to improve SOT outcomes.

### T_regs_ – targeted therapeutic strategies in SOT patients

**Figure 1:**
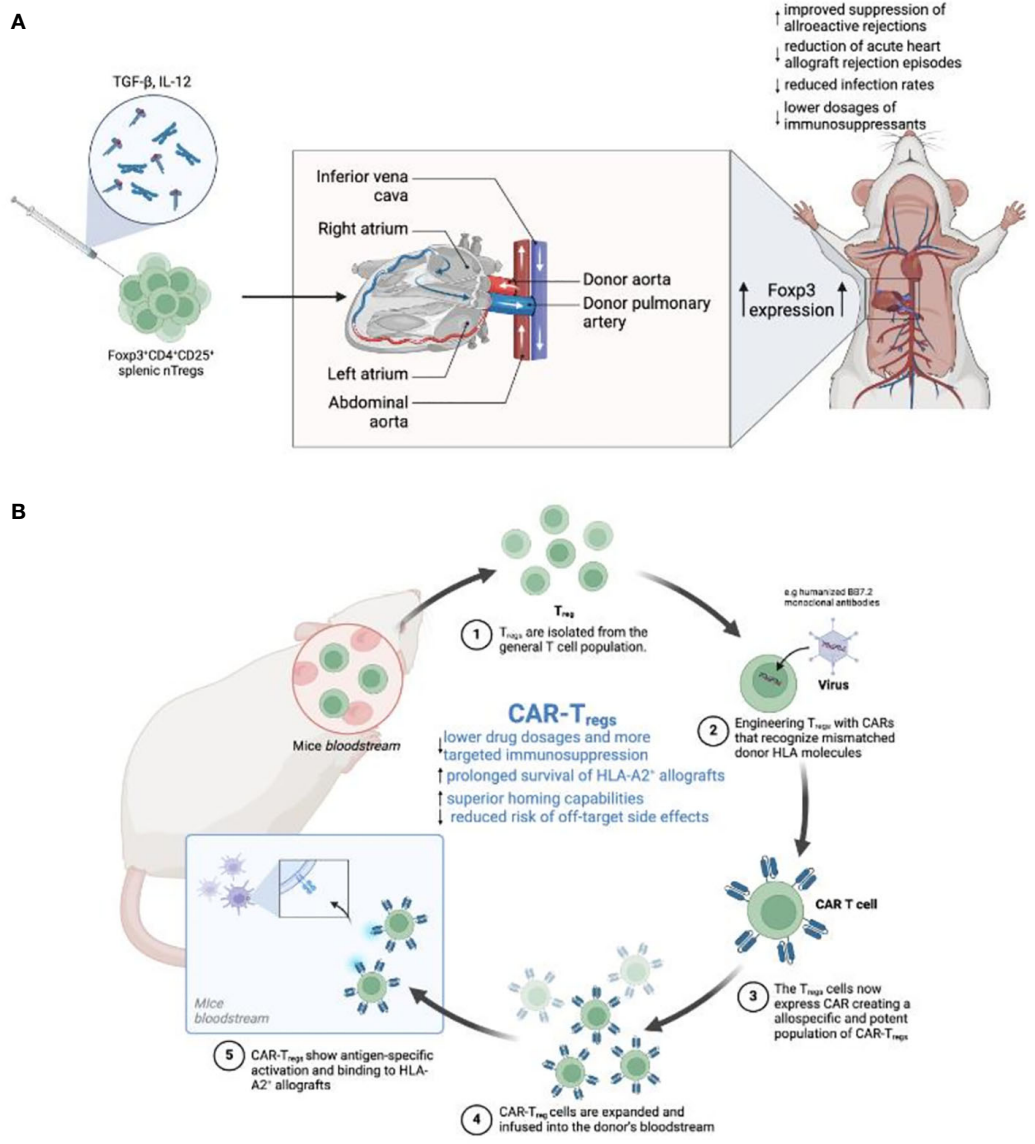
**(A)** Enhancing T_regs_ to promote tolerance in heart transplantation. This figure illustrates the sequential steps from the *in vitro* induction of T_regs_ using TGF-β and IL-12 cytokines to the maturation of Foxp3^+^CD4^+^CD25^+^ T_regs_. Additionally, the diagram provides a schematic representation of a human heart transplant, indicating the flow of blood through major vessels and heart chambers. This enhancement process is expected to improve transplant tolerance, reduce rejection rates, and potentially lower the need for immunosuppressive drugs (66). **(B)** Development of CAR-T_regs_ for targeted immunosuppression in a mouse model. The process begins with the extraction of T_regs_ from the mouse bloodstream, followed by their engineering to express CARs recognizing specific HLA molecules on transplanted tissues. The diagram depicts the binding of modified T_regs_ (CAR-Tregs) to HLA-A2+ allografts, showcasing antigen-specific activation. The expanded CAR-T_regs_ are then reintroduced into the mouse’s circulatory system. This targeted approach aims to minimize drug dosages, extend allograft survival, and reduce the risk of off-target side effects (67, 68).

T_regs_ have become crucial in the field of SOT, presenting new therapeutic possibilities for enhancing graft acceptance and decreasing the need for immunosuppressive medications (64, 65). Investigations into Foxp3^+^CD4^+^CD25^+^ splenic nT_regs_ have shown the critical importance of *ex vivo* expansion modifications in preserving Foxp3^+^ expression in SOT (66). Optimal Foxp3^+^ preservation was achieved through preconditioning CD4^+^CD25^+^ cells with TGF-β/IL-12, not only bolstering donor suppression but also effectively preventing acute heart allograft rejection (66) (**Figure 1A**). These findings laid the foundation for subsequent trials, such as the Autologous Polyclonal Expanded T_regs_ Adoptive Cell Therapy (TRACT) study (69). Patients who received autologous polyclonal expanded T_regs_ at 60-days post-transplant, following standard induction and daily maintenance immunosuppression, demonstrated an absence of infection or rejection for up to 2 years in a phase I clinical trial (69). Roemhild et al. further validated these outcomes by revealing that a combination of T_regs_ and conventional immunosuppressants enabled 90% of patients to maintain a low tacrolimus dose within the target range of 6-8 ng/mL over 48 weeks, with 72% displaying stable graft function when treated with nT_regs_ monotherapy (70).

Clinical trials have highlighted the impact of T_regs_ cell therapy on liver transplant recipients. Fueyo et al. conducted a phase 1 study assessing the safety and tolerability of polyclonal T_regs_ immunotherapy in patients undergoing liver transplantation from deceased donors (71). Results indicated that infusion of T_regs_ significantly reduced alloreactive T cell activity by over 20%, suggesting the potential for T_regs_ based therapy in liver transplantation (71). Further, Todo et al. demonstrated the potential of T_regs_-enriched cell products in liver transplantation, where patients received T_regs_ infusions after living donor liver transplantation, resulting in prolonged transplant survival of more than 15% (72).

“The ONE Study,” an EU-funded consortium, conducted a series of non-randomized, single-arm trials across several international sites to assess the safety and potential benefits of various regulatory cell-based medicinal products (CBMPs) in living-donor kidney transplant recipients (73). The study aimed to reduce reliance on general immunosuppression by employing CBMPs that included regulatory T cells (both polyclonal and donor-antigen reactive), tolerogenic dendritic cells, and regulatory macrophages. The trials demonstrated that this approach is feasible and safe, with a primary focus on the safety of regulatory CBMPs when combined with a reduced immunosuppressive regimen. Results showed similar rates of biopsy-confirmed acute rejection (BCAR) between the cell therapy groups and the reference group, which received standard immunosuppressive treatment. Furthermore, the study found that regulatory cell therapy was associated with fewer infectious complications, suggesting a potential benefit in reducing the burden of general immunosuppression in kidney transplant recipients (73). The ONE Study’s findings support the continued exploration of regulatory cell therapy as a therapeutic strategy in organ transplantation, with a focus on minimizing the adverse effects of long-term immunosuppression.

In another groundbreaking study, Tang et al. explored the use of donor-alloantigen-reactive regulatory T cells (darT_regs_) to induce graft-specific immunosuppression in liver transplant recipients, potentially reducing the need for broad immunosuppressive drugs (74). This phase I/II trial, named ARTEMIS (NCT02474199), aimed to test the safety and efficacy of darT_regs_ in maintaining stable liver function while allowing significant reduction in immunosuppression. Although the infusion of darT_regs_ in five out of ten participants who initiated immunosuppression withdrawal did not lead to adverse events, they faced challenges in manufacturing sufficient darT_reg_ doses and in achieving the primary efficacy endpoint—a 75% reduction in calcineurin inhibitor dose with stable liver function for at least 12 weeks (74). Only two participants managed to reach this endpoint, and mechanistic studies suggested darT_regs_ became dysfunctional after liver transplantation, affecting their efficacy. Mechanistic insights revealed a reduction in T_reg_ donor reactivity after liver transplantation, suggesting a need for further research to understand and potentially counteract T_reg_ dysfunction in this context (74). This study highlights several critical areas for future research, including overcoming manufacturing challenges, understanding the mechanisms behind T_reg_ dysfunction post-transplant, and exploring alternative strategies for inducing transplant tolerance.

A current phase I/IIa trial is investigating the safety and efficacy of inducing transient chimerism through a novel combination cell therapy in kidney transplant recipients without requiring myelosuppressive conditioning (75). Conducted in HLA-mismatched living donor kidney transplants, the approach involves administering *in vitro* expanded recipient T_regs_, donor BM cells, tocilizumab, and an anti-IL6R monoclonal antibody shortly after transplantation, with a regimen of thymoglobulin, belatacept, sirolimus, and steroids. Starting 6-months post-transplant, sirolimus and steroids are gradually withdrawn in stable patients, aiming for belatacept monotherapy. The control group receives a standard immunosuppressive regimen without T_regs_, anti-IL6R, or BM infusion (75).

Primary outcomes include safety measures and the assessment of donor chimerism within the first month post-transplant (75). Secondary outcomes focus on the frequency of acute and subclinical rejection episodes, graft function, and pro-tolerogenic immunomodulatory effects. This trial is driven by the hypothesis that recipient T_regs_ and donor BM infusion, combined with tocilizumab and a belatacept-based immunosuppressive regimen, can safely induce transient chimerism and a pro-tolerogenic immune environment in kidney transplant recipients (75). If successful, this innovative treatment could represent a significant advance in transplant immunomodulation, offering a safer alternative to current myelosuppressive chimerism-based tolerance protocols and potentially improving long-term transplant outcomes.

### T_regs_ for VCA surgery

Studies on the immunological dynamics during VCA graft rejection revealed a novel link between Foxp3 and IDO, an immune-regulatory enzyme that promotes an immunosuppressive environment (76). Over a period of 6 years, a total of 104 skin biopsies were obtained from bilateral hands of 3 human hand allograft recipients and assessed by hematoxylin-eosin histology and immunohistochemistry. The analysis revealed a temporally synchronized elevation of Foxp3 and IDO during graft rejection episodes (76). The simultaneous rise within the allograft suggested a coordinated function of Foxp3 and IDO to balance immune responses. This synchronization may provide a robust dual marker for detecting graft rejection and a potential lever for orchestrating targeted therapies to the rejection site.

T_regs_, with their intrinsic ability to modulate immune responses, hold promise in transplant immunology for their role in fostering graft tolerance and preventing rejection (77). However, despite their potential, there are numerous immunomodulatory limitations of T_regs_, such as the stability of T_regs_ in the inflammatory transplant environment (78). The inflammatory cytokines and post-transplantation milieu can lead to a phenomenon known as T_reg_ plasticity (79), where T_regs_ can lose their regulatory phenotype and potentially convert into pro-inflammatory effector T cells, thus contributing to graft rejection rather than prevention (79, 80). Furthermore, the scale-up of T_regs_ for therapeutic purposes has its challenges, including maintaining their suppressive function after expansion and ensuring that they retain their stability and functionality once infused back into patients (81). The *in vivo* survival, homing, and persistence of T_regs_ are critical factors that need optimization to ensure long-term graft tolerance (82).

Lastly, the regulatory pathways and mechanisms through which human T_regs_ exert their function are complex and not fully understood (83). This makes it challenging to predict and control their behavior within the immune system of a transplant recipient. The interactions between T_regs_ and other immune cells, such as dendritic cells, B cells, and T_effs_, are crucial for graft tolerance (84), but can also lead to unpredictable outcomes if not properly regulated (85). While T_regs_ present a significant therapeutic opportunity for inducing tolerance in SOT, their immunomodulatory limitations, such as lack of antigen specificity, stability, and complex regulatory mechanisms present hurdles that need to be addressed to fully harness their potential (86–88). Advances in genetic engineering, like the development of CAR-T_regs_, are promising strategies to overcome some of these limitations by enhancing specificity and control over T_reg_ activity.

### Chimeric antigen receptor-T cells – general cellular characteristics

CAR-T cells represent a class of adoptive cellular therapies exhibiting promising efficacy in the management of hematological malignancies like multiple myelomas (89). These specialized T cells are engineered with synthetic CAR constructs, comprising an extracellular antigen recognition domain derived from the single-chain variable fragment (scFv) of a monoclonal antibody, linked to intracellular signaling domains that induce T cell activation. This configuration allows CAR-T cells to directly bind to target antigens (TAAs) on the cell surface (67) (**Figure 1B**).

The structure of a CAR is segmented into several distinct components. The extracellular domain confers antigen specificity and is connected via a hinge region to a transmembrane domain that anchors the receptor in the T cell membrane (90). Intracellularly, the CAR possesses at least one signaling domains derived from the CD3ζ chain of the TCR complex and co-stimulatory molecules such as CD28, 4-1BB (CD137), or OX40 (CD134) (91, 92). The first generation of CARs only contained the CD3ζ signaling domain and, therefore, showed limited clinical efficacy due to inadequate T cell persistence and expansion (93). Second-generation CARs, which include an additional co-stimulatory domain, demonstrated improved T cell proliferation, cytokine production, and CAR-T cell survival (94). Third-generation CARs further augment the signal by incorporating two co-stimulatory domains, while fourth-generation CARs, also known as T cells redirected for universal cytokine-mediated killing (TRUCKs), are engineered to induce the secretion of transgenic cytokines upon antigen engagement, amplifying and sustaining the immune response (94). Different types of cells such as T_regs_, Natural Killer (NK) cells, and macrophages can be equipped with CAR constructs (95).

The effector functions of CAR-T cells are multifaceted, encompassing the secretion of cytotoxic granules such as perforin and granzyme (96), the production of inflammatory cytokines like IL-6, IFN-γ, TNF-α (97), and the induction of apoptosis in target cells (98). Upon antigen recognition, CAR-T cells undergo expansion and induce a broader immune response through the release of pro-inflammatory cytokines, which can recruit and activate additional immune effector cells (99, 100).

Turning to CAR-T_regs_, recent research has demonstrated the therapeutic potential of CAR-T_regs_ in dampening the pro-inflammatory responses of T_effs_ and demonstrating a stable phenotype even in a pro-inflammatory microenvironment (101, 102). Further, CAR-T_regs_ have been shown to secrete anti-inflammatory cytokines that promote immunotolerance (35). Research has shown promising potential in engineering CAR-T_regs_ to further exploit key pathways, such as TGF-β and IL-2 to improve the cellular stamina and therapeutic effectiveness of CAR-T_regs_ (64, 82, 90).

### CAR-T cells in SOT and VCA

To date, SOT and VCA research has mainly focused on CAR-T_regs_ by directing them against HLA (103, 104). By engineering T_regs_ with CARs that recognize mismatched donor HLA molecules, it is possible to create a population of T_regs_ that are both allospecific and potent, capable of homing to and protecting allografts from the host immune system with higher efficacy than polyclonal T_regs_ (103). Studies have demonstrated the viability of HLA-A2 specific CAR T_regs_, which show antigen-specific activation and demonstrate superior localization to HLA-A2 positive allografts, with the potential to substantially prolong allograft survival in pre-clinical humanized mouse models (104, 105).

The specificity of these CAR-T_regs_ enables them to outperform polyclonal T_regs_ in terms of suppression of the immune response against the transplant (103). This specificity is a crucial advantage because it may pave the way for lower dosages, yet more potent and targeted immunosuppression. In preclinical models, HLA-A2 specific CAR-T_regs_ have been able to prolong the survival of HLA-A2^+^ allografts (106–108). In these models, CAR-T_regs_ have demonstrated superior homing capabilities (103), preferentially migrating to HLA-A2^+^ graft sites when compared to polyclonal T_regs_ (106–108). Ultimately, this targeted homing ensures local and targeted immunosuppression, thereby reducing the risk of systemic immunosuppression and off-target side effects.

Specifically, Sun et al. described the development of CAR-T_regs_ tailored to identify the HLA-A2 antigen, which has been implicated in SOT transplant rejection (68). These cells, when engineered with the humanized BB7.2 monoclonal antibody, effectively migrated to HLA-A2 positive grafts while demonstrating reduced binding to other HLA alleles. Additionally, donor-specific (ds) CAR-T_regs_ administered to non-sensitized mice with HLA-A2^+^ skin grafts prolonged the graft lifespan by more than 30% (68). However, these findings were not reproducible in HLA-2 sensitized recipients, suggesting that previous immune exposure to the HLA-A2 antigen can influence the therapeutic efficacy of dsCAR-T_regs_.

MacDonald et al. took this approach further and designed HLA-A02 antigen-specific antibody (A2-CAR) CD8^+^ T_regs_, which effectively mitigated GvHD and skin rejection in NSG mice without inducing harmful cytotoxicity or altering the immunotolerant state of the endothelial cells (106). Of note, CAR-T_regs_ cells seem to be more efficient in inducing transplant tolerance compared to conventional T_regs_ (62, 109), with lower CAR-T_regs_ dosages (1.5×10^8^ CAR-T_regs_ versus 1×10^9^ T_regs_) showing similar immunosuppressive potency, when combined with T cell depleting agents (110). However, the application of CAR-T_regs_ is not without risks as complex genetic modifications have been implicated with neurologic and hematologic side effects (111, 112). Furthermore, the long-term stability of these cells, precise dosage requirements, and the potential for off-target effects remain areas of ongoing research (90).

Further preclinical research has shown the stability and persistence of CAR-T_regs_, which are critical determinants for long-term therapy success. However, the exact half-life of CAR-T cell therapeutics is still under investigation, with recent studies reporting CAR-T_regs_ infiltration in murine grafts for up to 40 days compared to conventional T_regs_ (113). An ongoing phase I/IIa clinical trial is set to provide more concrete data on the safety and efficacy of CAR-T_regs_ in living donor renal transplant recipients (NCT04817774)^120.^ While this trial may help determine the optimal dosing, frequency of infusion, and long-term stability of these cells in renal transplant patients, future research is needed to standardize CAR-T_reg_ for additional types of SOT and VCA transplants. Moreover, safety concerns such as tonic signaling, leading to T cell exhaustion and the phenotypic stability of T_regs_, remain to be further investigated (114).

Kauke-Navarro & Knoedler proposed an innovative strategy to mitigate transplant rejection, by harnessing and enhancing the immunoregulatory capabilities of T_regs_ (115). Recognizing the adverse effects associated with lifelong immunosuppressive therapy, the strategy emphasizes the bioengineering of T_regs_ to promote immune tolerance. These engineered T_regs_, potentially modified to express CAR constructs or specific T cell receptors, aim to target specific antigens, thereby minimizing off-target effects (115). The approach builds on the natural role of T_regs_ in maintaining immune homeostasis, including self-tolerance and antimicrobial resistance, and leverages their capacity for direct immunosuppression and modulation of the immune response. By focusing on antigen-specific T_regs_, the strategy seeks to provide a more targeted, effective means of preventing graft rejection while reducing the reliance on broad-spectrum immunosuppressive drugs. Further, they discuss the potential for employing advanced genomic editing techniques, such as CRISPR/Cas9, to enhance the specificity and safety of engineered T_regs_ (115).

Despite their potential, the utilization of CAR-T cell therapies in SOTs and VCA has a spectrum of limitations and challenges warranting consideration. Among these limitations is the protracted timeline inherent in the engineering and production of CAR-T cell products (116). The process encompasses several steps, beginning with the retrieval of patient-derived T cells, genetic manipulation to incorporate CAR constructs, *ex vivo* expansion of modified T cells, quality assessment procedures, patient conditioning, followed by infusion (117). This can take several weeks to months and is both costly and resource intensive (118), which may not align with the immediate needs of transplant patients who require rapid immune modulation to prevent rejection or manage complications like PTLD.

Unlike hematological malignancies where tumor cells express well-defined TAAs, the identification of suitable target antigens for CAR-T cell therapy in SOTs and VCA is more challenging (90). To our knowledge, there is limited research focused on this area, highlighting a significant gap in understanding and development of effective CAR-T cell therapies. Further, while CAR-T cell therapy offers the advantage of precise targeting of donor-specific HLA molecules, the potential for inadvertently targeting analogous antigens expressed on healthy tissues or vital cells presents a substantial challenge (119). Such off-target effects hold the potential to precipitate inadvertent tissue injury, autoimmune responses, or adverse events, thereby necessitating exhaustive preclinical evaluation of CAR-T cell specificity and safety profiles (120).

The protracted persistence and activity of CAR-T cells *in vivo* pose inherent risks of on-target side effects, such as sustained immune activation, CRS, and immune-related neuro-toxicities (121). Additionally, the prospect of CAR-T cell anergy or dysfunction over time remains a pertinent concern, as prolonged exposure to antigen stimulation may impair CAR-T cell functionality and constrain their efficacy in sustaining transplant tolerance or regulating alloimmune responses (122).

Moreover, the optimization of dosing regimens, frequency of administration, and long-term management of CAR-T cell therapies in transplant recipients necessitates further elucidation and standardization. The delineation of precise dosing and administration schedules for CAR-T cells may exhibit variability contingent on individual patient parameters, transplant modalities, and immunological status, thereby mandating personalized therapeutic approaches and monitoring. Given the potential variability, creating a standardized protocol and manufacturing process presents a challenge. Another concern is the potential interplay between CAR-T cells and standard immunosuppressive medications commonly used in transplant recipients introduces queries regarding pharmacological compatibility, drug-drug interactions, and the risk of additive or synergistic toxicities. Thus, we need a more comprehensive and in-depth understanding of the safety profile and long-term effects of CAR-T in transplant patients.

### Mesenchymal stromal cells - general cellular characteristics

MSCs can differentiate into different tissue types (cartilage, bone, fat, muscle) and have been shown to possess potent self-renewal capacities *in vitro* (123). They can be derived from various tissues including adipose tissue, umbilical cord, dental pulp, and bone marrow (124). Interestingly, MSCs do not express B7-1, B7-2, CD40, or CD40L, which are key co-stimulatory molecules for effective immune responses (125). The secretome of MSCs, a crucial paracrine regulator, has been demonstrated to impact fibrosis formation and wound healing through the release of vascular endothelial growth factor (VEGF), basic fibroblast growth factor (bFGF), nerve growth factor (NGF), transforming growth factor-beta (TGF-β), and keratinocyte growth factor (KGF) (126), while orchestrating chemotaxis via CCL5, CCL8, and CXCL12 (127). The MSC secretome also includes factors with anti-viral [indoleamine 2,3-dioxygenase (IDO), prostaglandin E2 (PGE_2_), interferon-γ (IFN-γ)] and anti-bacterial effects [hepcidin AMP (HAMP), lipocalin 2 (LCN2), and beta-defensin-2 (BD2)] (128). Studies have shown that MSCs can release factors such as hepatocyte growth factor (HGF) (129), tissue inhibitor of metalloproteinase 1 and 2 (TIMP-1 and TIMP-2) (130), and brain-derived neurotrophic factor (BDNF) (131), which all contribute to the MSC-induced anti-apoptotic effects on tissue cells.

The multifaceted role of MSCs in tissue regeneration and immune modulation is further underscored by their capability to expand T_regs_, linking their general cellular characteristics to potential therapeutic applications in immunotherapy (132). The absence of co-stimulatory molecules complements their role in immune regulation, particularly in the expansion of T_regs_ (133). This property is instrumental in preventing autoimmunity and in promoting immune tolerance, making MSCs a promising candidate for treating various autoimmune diseases and in transplant medicine (134). The secretome of MSCs plays a pivotal role in this context by mediating the immunosuppressive environment that fosters T_reg_ expansion (135). These interactions between MSCs and T_regs_ highlight a therapeutic synergy that leverages the inherent regenerative and immunomodulatory capacities of MSCs. This could pave the way for innovative treatments that combine tissue repair and immunological balance, addressing the complex needs of patients with autoimmune disorders or those undergoing organ transplantation.

### MSCs in SOT

**Figure 2:**
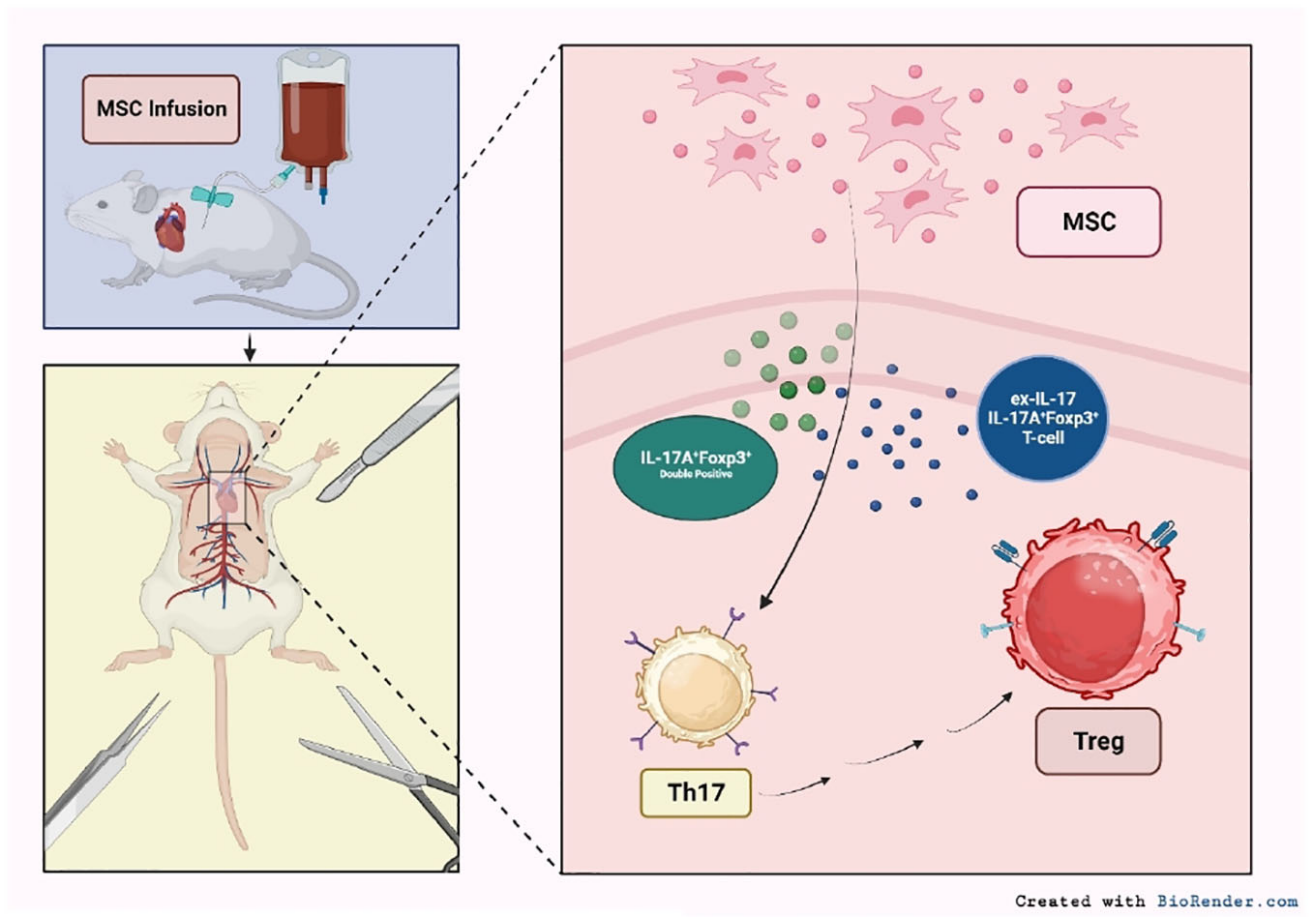
Mechanisms of MSC-mediated immune modulation in SOT. Infused MSCs interact with various immune cell populations, impacting Th17 and T_regs_ cell dynamics. The presence of MSCs further fosters the emergence of IL-17A^+^Foxp3^+^ cells and transitions IL-17-producing T cells to an IL-17A^-^Foxp3^+^ phenotype. This MSC-induced alteration facilitates the direct conversion of pro-inflammatory Th17 cells to anti-inflammatory T_regs_ (92).

The unique properties of MSCs position them as allies in modulating immune responses in SOT patients. Pre-clinical studies have highlighted the ability of MSCs to induce graft tolerance in both kidney and heart transplantation. For example, the presence of MSCs promoted the expression of IL-17A^+^Foxp3^+^ cells and IL-17-producing IL-17A^+^Foxp3^+^ T cells (136). These cells are potent counterparts to proinflammatory Th17 cells, which have been implicated with transplant rejection (137). Further, IL-17A^+^Foxp3^+^ subsets have been shown to drive T_regs_ expansion, ultimately suppressing rejection reactions and promoting graft tolerance (136). Overall, this MSC-induced transformation led to reduced inflammation, which correlated with extended graft survival (**Figure 2**) (136).

Building upon these pre-clinical observations, research has expanded into the nuanced roles of MSCs in immune modulation. A review by English et al. explored the effects of BM-MSCs on the innate immune system, particularly that BM-MSCs inhibited dendritic cell migration by promoting the transformation of mature dendritic cells to a pro-tolerogenic form (138). When dendritic cells were co-cultured with BM-MSCs and tumor necrosis factor alpha (TNF-α), there was a significant suppression of CD86 and major histocompatibility complex class II (MHCII), both pivotal drivers of immune activation (139). Meanwhile, expression levels of CCR7 and E-cadherin mRNA diminished, which have been shown to impact dendritic cell motility and adhesion (140). However, the effects of BM-MSCs on dendritic cells were transient: once dendritic cells were separated from the co-culture and reintroduced to TNF-α, they elicited an immune response while showing weak immunosuppressive effects (140). This work underscores the temporary nature of MSC-mediated effects, highlighting the intricacy of their therapeutic application. Such studies mark a crucial step forward, bridging fundamental research with the complex realities of immune interactions in a clinical setting.

A living-donor kidney transplant recipient was successfully induced into immune tolerance through the remote administration of autologous BM-MSCs (141). This groundbreaking case, believed to be the first of its kind, demonstrated that modulating the host’s immune system with MSCs enabled the complete withdrawal of maintenance immunosuppressive drugs while preserving optimal long-term kidney allograft function. The patient, a 37-year-old male with end-stage renal disease, received a kidney transplant from his father. Before transplantation, he was administered a dose of autologous BM-MSCs and underwent a low-dose rabbit antithymocyte globulin (RATG) induction therapy post-transplant. Maintenance immunosuppression was initially managed with CsA, MMF, and steroids, which were gradually tapered off. Eighteen-months post-withdrawal, the patient remained free from antirejection therapy with stable kidney function (141). This report is significant as it not only showcases the potential of MSCs to induce a sustained pro-tolerogenic immune environment in kidney transplant recipients but also illustrates the feasibility of safely discontinuing antirejection drugs. This achievement marks a significant advancement in transplant medicine, offering insights into the mechanisms of immune regulation and tolerance induction. The patient’s immune profile showed a high ratio of T_regs_ to memory CD8^+^ T cells, expansion of naïve and transitional B cells, and donor-specific T cell unresponsiveness, indicating a strong and lasting pro-tolerogenic milieu (141).

Additionally, Azevedo et al. explored the capability of MSCs to expand T_regs_ *in vitro*, aiming to support future clinical trials for immune tolerance therapies (132). Utilizing allogeneic BM-MSCs co-cultured with human peripheral blood mononuclear cells from healthy donors, the research found a significant increase in CD4^+^CD25^high^ Foxp3^+ CD127low^ T_reg_ cells after 14 days. This increase is attributed not to the proliferation of natural T_regs_ but to the conversion of conventional CD4 T cells into T_reg_-like cells in the presence of MSCs (132). These T_reg_-like cells demonstrated suppressive capabilities akin to natural T_regs_ and exhibited a DNA methylation profile closer to natural T_regs_, suggesting a stable conversion. The study points to mechanisms involving TGF-β and/or PD-1/PDL-1 expression in the induction process. This MSC-induced T_re_g population closely resembles natural T_regs_ in phenotype, suppressive ability, and methylation profile (132).

Interestingly, another study investigating the immunosuppressive properties of MSCs derived from fetal liver (FL) and adult BM, reveals a compelling differentiation in the capabilities of FL-MSCs (142). While both FL and BM-MSCs exhibit similar phenotypic profiles and differentiation capacities, FL-MSCs display significantly higher proliferative abilities and more potent suppression of CD4^+^ and CD8^+^ T cell proliferation. This suppression is accompanied by a notable increase in functional CD4^+^CD25^+^Foxp3^+^ T_regs_ when compared to BM-MSCs (142). The findings underscore the unique immunosuppressive activity of FL-MSCs, which not only directly suppress T cell proliferation but also indirectly enhance T_reg_ induction. This dual mechanism suggests FL-MSCs as a promising cell therapy source for treating immune-mediated diseases and preventing allograft rejection (142). The study highlights the advanced proliferative and immunosuppressive capabilities of FL-MSCs over adult BM-MSCs, emphasizing the potential of FL-MSCs in creating a favorable immunomodulatory environment for T_reg_ expansion, which could be pivotal for future clinical applications in immune tolerance.

Transitioning to clinical investigations, the safety and therapeutic potential of umbilical cord-derived MSCs (UC-MSCs) were studied in a cohort of 27 liver transplant patients (143). Upon receiving a single infusion of UC-MSCs (1x10^6^/kg body weight), the patients demonstrated a pronounced expansion of peripheral T_regs_ over 4 weeks. Notably, there was an elevation in the levels of PGE2 and TGFβ-1, key mediators for attenuating immune responses and fostering tissue repair. Histological analysis post-infusion showed a significant reduction in the total rejection activity index (RAI) scores in 43% of patients (143). Further findings from this study revealed that UC-MSC treatment led to marked declines in liver damage indicators such as alanine transaminase (ALT), aspartate aminotransferase (AST), and total bilirubin (TBIL). Additionally, there was an upregulation in T_regs_, a downregulation in Th17 cells, and a suppression of CD4^+^ T cell activation, as evidenced by decreased HLA-DR expression. Plasma levels of TGF-β1 and PGE2, both integral to immunoregulation, substantially increased 4-weeks post-infusion (143). These findings underscore the potential therapeutic benefits of UC-MSCs, indicating not only their safety but also their effectiveness in alleviating liver damage and modulating immune responses in liver transplant recipients with acute rejection.

Reinders et al. conducted additional clinical investigations to assess the effectiveness and safety of MSC therapy in renal transplantation (144). They carried out a randomized, prospective, single-center, open-label trial to compare the outcomes of post-transplant MSC infusion with those of a control group receiving standard tacrolimus doses.

This investigation, designed to address the need for immunosuppressive regimens that effectively prevent allograft rejection without compromising renal function or leading to significant side effects, involved 70 patients who were initially randomized, with 57 ultimately included in the final analysis (29 in the MSC group and 28 in the control group) (144). The study’s primary endpoint was the quantitative assessment of interstitial fibrosis from biopsies taken at 4 and 24 weeks after transplantation, aiming to discern the potential benefits of MSC therapy in mitigating fibrosis compared to conventional immunosuppression strategies. Secondary endpoints encompassed a range of critical outcomes, including rates of acute rejection, graft loss, patient survival, renal function metrics, adverse events, and immunological responses, particularly focusing on the regulatory T cell populations as indicators of the immunomodulatory effects of MSC therapy (144). The results revealed that MSC therapy, combined with early tacrolimus withdrawal, did not demonstrate a statistically significant benefit in reducing interstitial fibrosis compared to the control group (p=.014). Renal function remained stable across both cohorts, with only one instance of acute rejection documented in the MSC group. Interestingly, regulatory T cell numbers were significantly higher in the MSC group at the 24-week mark, suggesting a potential immunomodulatory benefit of MSC therapy that warrants further investigation (144).

The Neptune study, a phase I single-center trial, explored the safety and feasibility of using allogeneic MSCs in renal transplantation, focusing on a novel approach of HLA-selected allogeneic MSC therapy combined with adjusted immunosuppression (145). This nonrandomized, prospective study involved ten patients who underwent renal transplantation from living donors. 6-months post-transplantation, these patients received two doses of 1.5×10^6^/kg allogeneic MSCs. The immunosuppressive regimen included a reduction in tacrolimus to a trough level of 3 ng/mL, supplemented with everolimus and prednisone (145). The primary goal was to assess safety, particularly looking at biopsy-proven acute rejection (BPAR) and graft loss over a 12-month period following MSC infusion. Remarkably, the study reported no instances of BPAR or graft loss, and renal function remained stable throughout the follow-up period. Immune monitoring showed no significant alterations in T and B cell populations or plasma cytokine levels post-MSC infusion (145). Interestingly, one patient had pre-existing donor-specific antibodies (DSAs) against the MSCs, indicating sensitization prior to infusion. However, this did not impact the overall safety profile or renal function. The MSCs were carefully selected to prevent repeated HLA mismatches, minimizing the risk of an immune response against the allograft (145). This pioneering trial demonstrates that administering HLA-selected allogeneic MSCs, in conjunction with a tailored immunosuppressive regimen, is safe within the first year after renal transplantation. It lays the groundwork for further research into the efficacy and long-term outcomes of third-party MSC therapy in transplant medicine, offering a potential new avenue for enhancing graft survival and patient outcomes.

Subsequent research efforts have aimed to further elucidate the safety and efficacy of MSC therapy in kidney transplantation (146). An open-label phase I-II clinical trial involving 20 kidney transplant recipients who received kidneys from deceased donors were divided into two groups: one group received a single infusion of third-party bone marrow-derived MSCs (approximately 2-3 million MSCs per kilogram) on day 3 post-transplant, while a concurrent control group did not receive MSC therapy. The study monitored patients for adverse effects related to MSC infusion, opportunistic infections, acute rejection incidents, renal function, and the development of antibodies against MSCs or shared kidney-MSC HLA antigens (146). One of the key findings was the absence of significant adverse effects at MSC injection, although one participant with a history of cardiac disease experienced a non-ST-elevation myocardial infarction shortly after the MSC infusion. The incidence of opportunistic infections and acute rejections did not differ significantly between the MSC-treated group and the control group, suggesting that MSC therapy did not increase the risk of such complications (146). A notable observation was the early improvement in estimated glomerular filtration rate (eGFR) among MSC-treated recipients, reaching 48.6 ml/min/1.73m2 by day 7 post-transplant, compared to 32.5 ml/min/1.73m2 in controls and 29.3 ml/min/1.73m2 in the overall cohort of kidney transplant recipients at the same institution (146). This early improvement suggests a potential nephroprotective effect of MSC therapy, although no significant difference in eGFR was found at the one-year mark. Immunologically, the MSC-treated group exhibited increased frequencies of T_regs_ at day 30 post-transplant, without significant changes in B cell frequencies (146).

This observation points towards an immunomodulatory benefit of MSC therapy, possibly contributing to a more tolerant and less inflammatory post-transplant environment. Moreover, four participants in the MSC-treated group developed antibodies against MSC or shared kidney-MSC HLA antigens, with only one showing a mean fluorescence intensity (MFI) greater than 1500, indicating a relatively low level of immunization against the infused MSCs (147). The study concludes that a single infusion of third-party MSC following kidney transplantation is safe and feasible, with an observed cardiac event of unclear relation to the intervention. The MSC therapy was associated with improved early allograft function and increased regulatory T cell proportions, suggesting potential immunomodulatory benefits (148).

Expanding upon this, in another prospective, randomized control trial, 159 end-stage renal disease patients undergoing ABO-compatible, cross-match–negative kidney transplants received kidneys from living-related donors (148). The intervention involved inoculating patients with marrow-derived autologous MSCs (1-2 × 10^6^/kg) at the time of kidney reperfusion and again two weeks later. The study was designed to assess whether autologous MSCs could replace antibody-based induction therapy, traditionally combined with CNIs, to reduce acute rejection rates while mitigating the risks of opportunistic infections and toxic effects associated with CNIs (15). The participants were divided into three groups: one received autologous MSCs plus standard-dose CNIs, another received MSCs plus low-dose CNIs (80% of the standard dose), and the control group was treated with anti–IL-2 receptor antibodies plus standard-dose CNIs (148). The primary outcome measured was the incidence of acute rejection and renal function, as indicated by the eGFR, within the first-year post-transplant. Secondary measures included patient and graft survival rates and the incidence of adverse events. The findings revealed compelling advantages of using autologous MSCs over traditional induction therapies (148). Acute rejection within six-months post-transplant was significantly lower in both MSC-treated groups compared to the control group, with 7.5% and 7.7% in the MSC groups versus 21.6% in the control group. Notably, none of the patients in the MSC groups experienced glucocorticoid-resistant rejection, which was observed in 7.8% of the control group (148). Furthermore, renal function recovery was faster among the MSC-treated patients, demonstrated by significantly increased eGFR levels in the first month post-surgery compared to the control group. The study also noted a reduced risk of opportunistic infections in the MSC-treated groups, highlighting the immunomodulatory benefits of MSC therapy (148). Patient and graft survival rates at 13 to 30 months were comparable across all groups, emphasizing the safety and feasibility of autologous MSC therapy in kidney transplantation. The incidence of adverse events was lower in the MSC groups, particularly regarding opportunistic infections, further underscoring the potential of MSC therapy to enhance transplant outcomes while minimizing the risks associated with standard immunosuppressive protocols (148).

Of note, the therapeutic utility of MSCs in SOTs depends on multiple factors including timing, the expansion behavior of MSCs, cell-cell interactions, and their transient effects on other immune cells (149, 150). Studies have shown that the timing of MSC administration could significantly influence their immunomodulatory abilities and transplant outcomes (147, 151). In fact, participants who were administered MSCs prior to kidney transplantation exhibited lower serum creatinine levels, ranging between 1.35-0.97 mg/dL and 1.37-1.49 mg/dL, in contrast to those who received post-transplant infusions, where levels ranged between 1.83-2.27 mg/dL and 2.38-2.21 mg/dL (147). Serum creatinine is a marker often used to evaluate kidney function—elevated levels can indicate impaired kidney function or kidney disease (147). Paralleling this finding, Casiraghi et al. have shown that introducing MSCs prior to transplantation could elevate their immunosuppressive effects, which may directly suppress acute graft complications (152).

Besides transplantation timing, the cellular profile of MSCs has been demonstrated to influence transplant outcomes. In a study using a murine kidney transplant model, mice underwent a single MSC infusion (0.5×10^6^ cells) either before or after the transplant procedure (153). The post-transplant group (n=7) exhibited higher blood urea nitrogen (BUN) levels than the pre-transplant mice, pointing to possible renal dysfunction. Further, the authors observed significantly higher levels of neutrophils, complement C3 deposition, interleukin 6 (IL-6), and TNF-α in the post-transplant group on days 2 and 7 following MSC-infusion, indicating a prolonged inflammatory immune response (153). Conversely, pre-transplantation MSC administration resulted in an upregulation of T_regs_ and extended graft survival by more than 60 days. Furthermore, the study shed light on the MSC homing behavior. After infusion, MSCs first positioned themselves in the spleen and later migrated to the allograft (153). The migration process may influence immune modulation, potentially promoting graft tolerance and impacting cell-cell interactions crucial for graft acceptance or rejection via the T_regs_ axis.

### MSCs for VCA

Allogeneic BM-MSC transplantation, either co-transplanted with vascularized bone marrow or alone in a hemi-facial allotransplant model, significantly extended the survival of pig limb allografts when combined with irradiation and cyclosporine A (CsA) treatment (154). Moreover, repetitive high-dose BM-MSC treatment (>1x10^6^ cells) extended the survival of pig hemi-facial transplant recipients by over 20 days, even in the absence of conditioning therapy; these grafts experienced only mild rejection (Grade I to II), which improved under CsA treatment (155). The beneficial effects of BM-MSCs on rejection grades were also associated with upregulated IL-10 expression and the induction of T_regs_, underscoring the dampening effects BM-MSCs on the recipient immune response (156).

**Figure 3:**
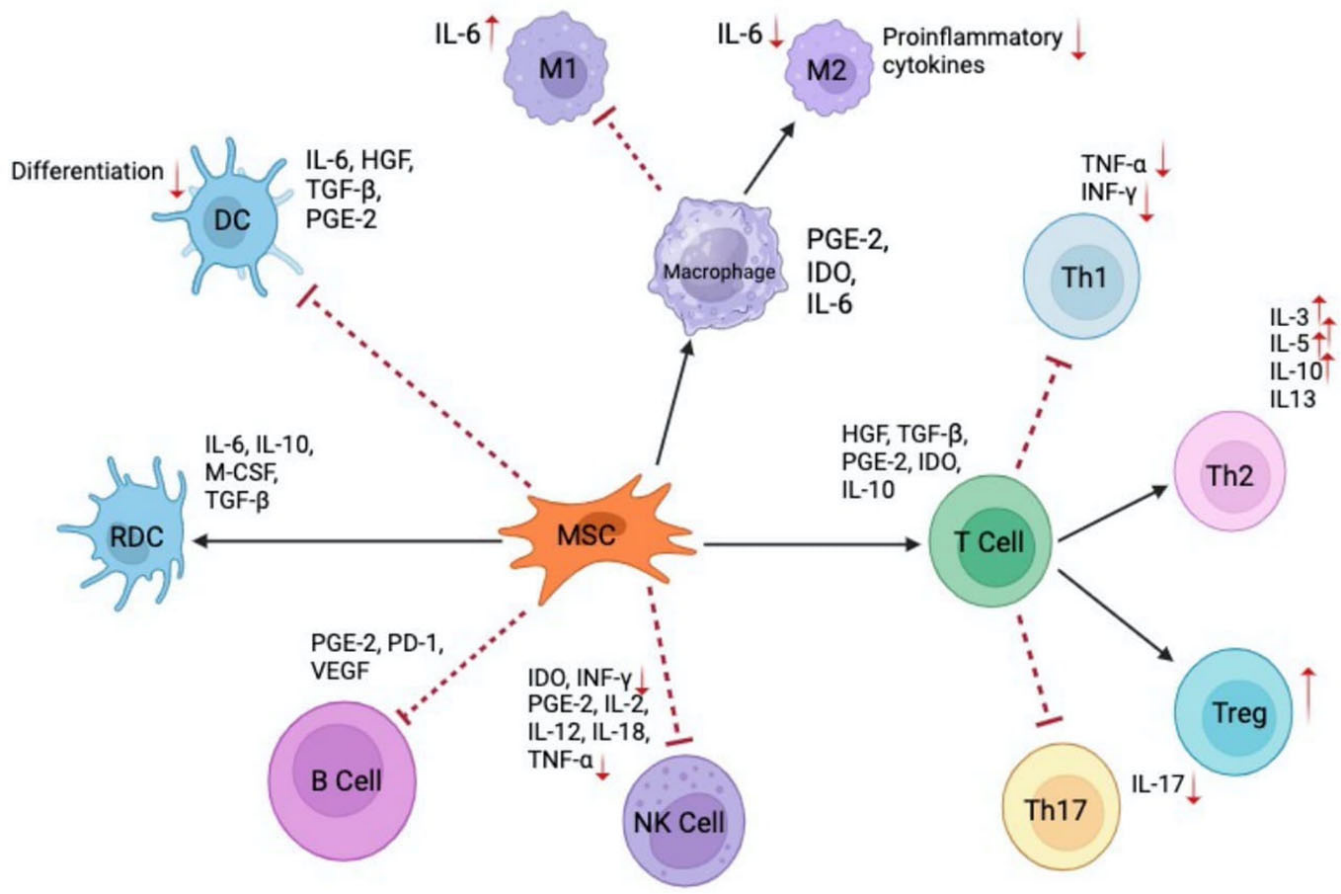
Interactions of MSCs with immune cells and their mediated cytokines. MSC illustrates the role in modulating various immune responses through cytokine secretion and direct interactions with multiple immune cell types. Dendritic cells (DC) and regulatory dendritic cells (RDC) and their differentiation modulated by cytokines such as 1L-6, HGF, TGF-β, and PGE2 (131). Macrophages (MI and M2) showcase the polarization of macrophages into Ml (pro-inflammatory) and M2 (anti-inflammatory) phenotypes in the presence of 1L-6 and other modulating factors. MSCs play a role in this differentiation through the release of cytokines like PGE-2, IDO, and 1L-6 (132). T Cells illustrates the various T cell subsets - Th1, Th2, Treg, and Th17, and their interactions with MSCs. Cytokines like HGF, TGF-β1, PGE-2, 1DO, and IL-10 play a role in modulating these T cell responses (133). MSCs impact B cells and natural killer (NK) cells through the secretion of mediators such as PGE-2, PD-1, VEGF for B cells and IDO, INF-γ, PGE-2, IL-2, IL-12, IL-18, and TNF-α for NK cells (134).

In another approach, Kuo et al. highlighted the effects of repeated AD-MSCs doses in promoting tolerance in hind limb rat VCA model and driving the proliferation of tolerance-inducing CD4^+^CD25^+^Foxp3^+^ T_regs_ (157). Conversely, a single dose of syngeneic AD-MSCs (2x10^6^ cells) was shown to induce tolerance in a rat VCA model, even in the presence of donor-recipient MHC mismatch when administered preoperatively in combination with CsA and anti-lymphocyte globulin (157). Currently, there is no consensus on the dosing and timing of MSC administration in VCA protocols. Experimental studies have shown beneficial results with MSC dosages ranging from 5×10^5^ to 5×10^7^ cells per kg body weight, administered at various time points relative to transplantation (151). Overall, future research work is needed to standardize MSC-based therapies for VCA and SOT patients and balance the various cellular interactions of MSC populations. The complex cellular and cytokine network involving MSCs is shown in **Figure 3**.

### Cotransplantation of vascularized bone marrow in VCA surgery

Another cellular strategy to mitigate alloreaction and improve transplant acceptance is based on the hypothesis that the co-transplantation of vascularized bone marrow (VBM) compartments (mandible, ulna, radius) generates a continuous source of donor-derived hematopoietic stem cells (158). In fact, this approach has shown promising potential to modulate the recipient’s immune system in different animal models. In one notable study, researchers investigated the effects of co-transplantation in rats, dividing them into groups where Group I received syngeneic transplants as a control, and Groups II-V underwent allogeneic transplants with receiving antilymphocyte globulin (5 mg) and tacrolimus (1 mg/kg), along with total body irradiation of 0, 600, 400, and 200 cGy respectively, prior to transplantation (159). On day 0, each Lewis rat in Groups II-V received a Brown Norway hind-limb osteomyocutaneous flap. The presence of different donor hematopoietic cell lineages in recipients’ blood was assessed on days 15, 30, 60, 90, 120, and 150 post-transplantation using flow cytometry. The researchers showed that allotransplanted hind-limb flaps in Groups III, IV, and V led to chimerism and donor multilineage hematopoietic cells, with acceptance rates of 37.5%, 16.7%, and 0% respectively (159).

Building on these findings, Barth et al. developed a nonhuman primate model of facial segment allotransplantation in cynomolgus monkeys (155). Heterotopically transplanted facial segment VCA with VBM (n=4) treated with tacrolimus and MMF demonstrated prolonged rejection-free survival between 205 and 430 days, compared to VCA without VBM that demonstrated early rejection episodes and graft loss by postoperative days 7–15^168^. Further investigation in a rat model that combined hemiabdominal wall and hindlimb osteomyocutaneous flaps with VBM transplantation revealed that a subset of recipients showed long-term allograft survival, developed donor-specific tolerance, and exhibited significantly higher levels of peripheral chimerism (160). This study also pointed out that a balanced ratio of allograft to VBM mass is crucial for optimizing chimerism and enhancing graft survival. The authors found that 3 of 8 VBM recipients showed long-term allograft survival of more than 100 days and developed donor-specific tolerance. Further, VBM groups demonstrated significantly higher peripheral chimerism, analyzed through flow cytometry. Interestingly, the authors reported that higher ratios of allograft to VBM mass correlated with lower levels of chimerism and reduced graft survival, underscoring the need for balancing VBM against VCA transplant weight (160).

However, translating these animal model successes to human clinical practice has been challenging. To date, evidence of long-term donor chimerism in patients undergoing hand, forearm, arm, or face transplantation, even with the inclusion of facial bone, is limited (161). There has been one reported case of a face transplant patient who, after receiving a mandible co-transplantation along with an infusion of hematopoietic stem cells, exhibited transient chimerism of CD34+ T-cells (162).

Beyond the cotransplantation of VBM, infusion of donor BM cells have shown positive outcomes in animal models (163, 164). Based on these experimental findings, Schneeberger et al. established the Pittsburgh Protocol (161). The authors enrolled 5 patients who either received a bilateral hand (n=2), a bilateral hand/forearm (n=1), or a unilateral (n=2) hand transplant. All patients were treated with alemtuzumab and methylprednisolone for induction therapy, followed by tacrolimus monotherapy as maintenance treatment. On day 14, patients received an infusion of donor BM cells isolated from the patients’ vertebral bodies. Immunomonitoring through flow cytometry revealed transient moderate levels of donor-specific antibodies, adequate immunocompetence, but no peripheral blood chimerism (161). While the authors concluded that the Pittsburgh Protocol represented a safe and well tolerated approach for low-dose tacrolimus monotherapy following upper extremity transplantation, their findings remain to be confirmed in long-term follow-up studies (161). Overall, the clinical effects of donor-derived bone marrow cells, either infused or as VBM cotransplant, still stand in sharp contrast to the promising *in vitro* and *in vivo* data, therefore warranting future research work.

### Regulatory myeloid cells – basic cellular characteristics

RMCs, including macrophages, dendritic cells, and MDSCs, play pivotal roles in both immune regulation and tissue repair (165). They originate from diverse sources, ranging from the embryonic yolk sac to hematopoietic stem cells and bone marrow-derived monocytes (166). Beyond their immune-modulating functions, RMCs are instrumental in tissue regeneration, particularly within the TNFR1/TNF-α and IL-8 signaling pathways (165). They exhibit clinical promise in mitigating excessive inflammation through the release of IL-10 and TGF-β, orchestrating angiogenesis, and facilitating tissue remodeling via the modulation of pro-inflammatory and anti-inflammatory cytokines at different stages (34). This dual capacity of immune suppression and tissue repair positions RMCs as a promising avenue for advancements in transplantation research. A deeper understanding of RMC biology and function could unveil strategies to optimize VCA outcomes, bridging the gap between rejection and tolerance.

RMCs encompass subsets like M_regs_, DC_regs_, and MDSCs. M_regs_, akin to M1 macrophages, differentiate from monocytes in response to M-CSF and IFN-γ (167). They are characterized by their secretion of critical cytokines, including IL-10 and TGF-β (168). Conversely, DC_regs_ play a central role in immune regulation by promoting T_regs_ development, balancing T cell responses and inducing T cell apoptosis, particularly in lactate-enriched microenvironments (49). MDSCs, known for their potent immunosuppressive capabilities, comprise multiple subtypes, each with distinct functional attributes (169). These subtypes, ranging from polymorphonuclear (pmnMDSC) to monocytic (mMDSC) and early-stage (eMDSC) cells, employ various mechanisms for immune suppression (170). Such mechanisms include cytokine secretion, depletion of essential amino acids like arginine and tryptophan, and the production of reactive oxygen and nitrogen species. Additionally, they demonstrate a dual role by inhibiting the actions of T cells and NK cells, while enhancing the functions of T_regs_ (171).

### RMCs in SOT patients

Researchers investigating the optimization of SOT have explored cellular mechanisms influencing rejection and tolerance. Central to this inquiry is the role of M_regs_, which have been associated with significant advancements in graft acceptance and reduction of immunosuppression (172). Conde et al. identified critical determinants and signals altering RMC response following SOTs (173). They observed that the regulation of dendritic cell C-type lectin receptor (DC-SIGN) in macrophages through IFN-γ and colony stimulating factors (CSFs) led to an upregulation in CD4^+^ T cell expansion and a decline in CD8^+^ T cell populations. This effect can be attributed to either the manipulation of Fc**γ**-receptor (FcγR) ligation on activated macrophages or the modulation of DC-SIGN^+^ macrophages through IFN-**γ** and CSF1 (173). Further exploring this pathway, IFN-**γ** and macrophage colony stimulating factor (M-CSF) were crucial in generating M_regs_ that suppress polyclonal T cell proliferation via an inducible nitric oxide synthase (iNOS)-dependent mechanism (174).

To manipulate RMCs, researchers used double-stranded oligodeoxynucleotides (ODNs) to decrease nuclear factor kappa B (NF-κB)-driven gene activity and IL-4 production (175). Combining these modified cells with recombinant adenovirus (rAd) vectors created a hybrid termed rAd CTLA4-Ig/NF-κB ODN DCs. Compared to untreated allografts, these hybrid cells led to a significant 71-day extension in graft survival and decreased inflammation levels (measured by Th1 and Th2 cytokine levels) at a concentration of 2x10^6^ cells per mouse (175). In parallel with these findings, administering a combination of 2x10^6^ MDSCs and 3 mg/kg of rapamycin on postoperative days 0, 2, 4, and 6, resulted in markedly extended graft survival in mice with heart transplants (172). Interestingly, M_regs_ monotherapy led to significantly shorter graft survivals compared to the combination treatment (170).

Beyond their direct impact, M_regs_ can also modulate the activity of other immune cells. For instance, they have been shown to induce the conversion CD4^+^ T cells into M_regs_-induced T_regs_ (miT_regs_), which release IL-10, thereby promoting the proliferation of T_regs_ populations (176). This distinct cell subset is characterized by butyrophilin like 8 (BTNL8) gene expression (177). Moreover, M_regs_ can stimulate the expression of T cell immunoreceptor with Ig and ITIM domains known as TIGIT^+^iT_regs_ populations (177). Additionally, M_regs_ can secrete PGE2 serving as a crucial mediator for macrophages by promoting anti-inflammatory cytokine synthesis while suppressing pro-inflammatory molecules like TNF-α and IL-12 (178).

On the dendritic cell front, Cai et al. created immature DC_regs_ derived from induced pluripotent stem cells (iPSCs) (179). They showed that a dosage of 1x10^6^ iPS-DCregs sustained permanent allograft tolerance in a murine cardiac transplant model (179). In a phase I/II trial involving 12 liver transplant recipients, researchers discovered that injections of donor-derived DC_regs_ decreased T cell proliferation, increased T_regs_ populations, and balanced the IL-10-to-IL-12 ratio post CD40Ld activation, ultimately driving transplant tolerance (84). Histological samples from study participants (n=10) showed a programmed death ligand-1 (PD-L1) to CD86 ratio exceeding 2.5, which has been proposed as another marker of transplant tolerance (84).

The role of MDSCs in regulating immune responses is well documented. They suppress the proliferation of T_effs_ without affecting their activation, mediated by the iNOS enzyme (180). Furthermore, there is an association between MDSCs and increased T_reg_ levels, alongside decreased IL-17 production (136). Additional research has confirmed the correlation between MDSCs and T_reg_ accumulation, with a specific MDSC subset, CD11b^+^CD33^+^HLA^-^DR^-/lo^, promoting the expansion of naive CD4+Foxp3+ T_regs_ *in vitro* (181). This expansion also correlated with a rise in cytokines such as tumor growth factor beta-1 (TGF-β1) and IL-10 (41). Interestingly, the immunosuppressive ability of this MDSC subset was only observed in renal transplant recipients but not in non-transplant controls (182). They further reported that, among MDSCs, CD14^+^ mononuclear (M)-MDSCs exhibited more potent immunosuppressive effects than CD14^+^Granulocyte-like MDSCs (G-MDSCs), even though both subsets increased in transplant recipients (182).

Immediate post-transplant introduction of MDSCs has been associated with prolonged graft survival of more than 19 days when combined with anti-CD154 monoclonal antibodies (183). However, MDSC therapy has also been implicated with increased risk of malignancies. Utero-Rico et al. examined 229 renal transplant recipients, focusing on the relationship between certain myeloid cells and cancer risk (184). The results indicated that high M-MDSC levels, especially surpassing >179.2 per microliter on day 14 post-transplant, correlated with a sevenfold increase in all-types cancer risk theoretically by predisposing to an autoimmunoreactive environment (184). Interestingly, prolonged elevated M-MDSC levels were also correlated with stronger immunosuppressive action and reduced antioxidant activity (184). Thus, while MDSCs seem promising for early post-transplant care, their extended application warrants further investigation and closer perioperative monitoring.

## Conclusion

Cellular therapies have emerged as promising alternatives to current immunosuppressive drugs in transplantation. Their versatility and specificity offer an opportunity to precisely regulate the immune system, potentially extend graft longevity, and reduce the dosages of conventional immunosuppressive regimens. These therapies, representing a fusion of immunological insights and bioengineering innovations, mark a paradigm shift from broad-spectrum immunosuppression to a tailored immunomodulatory strategy, potentially minimizing the adverse effects associated with conventional regimens.

Within the expanding framework of SOTs and VCAs, cellular therapies are illuminating pathways towards optimizing perioperative care in transplantation. From augmenting the immunoregulatory capabilities of T_regs_, to leveraging the potent immunosuppressive effects of MSCs, and capitalizing on the distinctive capabilities of CAR-T cells, these methodologies offer a sophisticated approach to inducing immune tolerance, averting graft rejection, and facilitating robust graft integration. Furthermore, certain microenvironmental factors implicated with poor transplant function, such as elevated lactate levels, could be leveraged to potentiate the immune-modulating properties of these cell therapeutics (49). The current body of knowledge suggests that the multifaceted immunomodulatory roles of cellular therapies may advance perioperative patient management following SOTs and VCAs.

As discussed, the combination of various cell types may strengthen the therapeutic effects by compensating for cell-specific limitations and leveraging cell-specific tolerance-inducing capacities. The transplant microenvironment and timing of cell therapeutics represent understudied but promising angles for advancing peri-transplant care (147, 149–151). Broadening the pool of cell subsets with immunosuppressive effects could further pave the way towards more individualized cell therapeutics. While these strategies carry promising potential for both SOT and VCA, future VCA-specific trials are needed to bridge the research gap between SOT and the emerging field of VCA surgery.

Despite these promising advances, the clinical translation of cellular therapies into routine transplantation care faces significant hurdles. The challenges span the need for standardized protocols for cell isolation, expansion, and infusion, to a deeper understanding of the molecular and cellular mechanisms underpinning graft rejection and tolerance to further advance cellular therapeutics. Moreover, the identification of reliable biomarkers for monitoring therapeutic efficacy, understanding the optimal timing for therapy administration, and unraveling the synergistic potential between cellular and conventional therapies remain critical areas for future research to determine the most effective ratio of cellular to conventional immunosuppressive regimens.

Tr1 cell therapy has shown promising potential in transplantation to improve graft acceptance and patient outcomes, representing a significant step toward more targeted and less toxic immunomodulation strategies (37, 42). However, the need for specific Tr1-cell biomarkers, enhanced *in vitro* expansion techniques, and a deeper understanding of their regulatory mechanisms pose challenges that future research must address to fully harness the therapeutic potential of Tr1 cells in clinical practice. The innovative concept of incorporating Tr1 cells into CAR constructs could further refine their specificity and functionality (38, 47), representing an exciting frontier for future therapeutic development in this field.

As CAR-T cell therapies emerge as promising and innovative modalities for enhancing transplant acceptance and reducing reliance on conventional immunosuppression, their integration into transplant care paradigms requires careful consideration and mitigation of multifaceted challenges and limitations (62, 68, 103–115, 185). Rigorous preclinical investigations, well-designed clinical trials, and ongoing research is imperative to comprehensively elucidate the therapeutic potential, safety profiles, and outcomes of CAR-T cell therapies in SOTs and VCA. Additionally, the careful selection of target antigens is critical to minimizing the risk of adverse events and ensuring the safety of CAR-T cell-based treatments.

Further, the complex pharmacological interplay between the patient’s standard immunosuppressants and CAR-T cells remains to be fully elucidated. Personalized therapies, such as CAR constructs, offers both benefits and limitations. While personalized therapy holds the potential to tailor treatments to individual patients, providing more targeted and effective interventions, it also requires time for customization that off-the-shelf therapies do not. As medicine increasingly adopts personalized therapeutic approaches, balancing the benefits of tailored treatments with the time required for their development becomes essential.

In this comprehensive review, we analyzed the dual impact of cellular therapies on SOTs and VCA. Overall, this advanced understanding of cellular therapies could lead to more targeted perioperative treatment strategies in SOTs and VCAs. While these therapies have demonstrated positive clinical outcomes in transplant patients, their standardized integration into transplant care requires further investigation in future trials. This review underscores the potential of cellular therapies to revolutionize transplant surgery, emphasizing the need for rigorous scientific inquiry and systematic evaluation to validate their efficacy, safety, and feasibility on a broader scale. Moving forward, continued research efforts are essential to elucidate the precise role of cellular therapies in transplant medicine and to address the logistical and ethical challenges associated with their widespread adoption.

## Author contributions

LK: Conceptualization, Writing – original draft, Writing – review & editing. JD: Writing – original draft, Writing – review & editing. FD: Writing – original draft. NT: Writing – original draft. SK: Writing – original draft, Writing – review & editing. RR: Data curation, Writing – review & editing. KS: Writing – original draft. TE: Writing – original draft. SM: Writing – original draft. FF: Writing – original draft. KK: Writing – original draft. AP: Writing – original draft. JI: Writing – original draft. A-FS: Writing – original draft. ST: Writing – review & editing, Supervision, Validation. SH: Writing – review & editing. BP: Conceptualization, Project administration, Supervision, Writing – review & editing. MK-N: Conceptualization, Project administration, Supervision, Writing – original draft, Writing – review & editing.

## Glossary

**Table:**

A2-CAR	HLA-A02 Antigen-specific Chimeric Antigen Receptor
AD-MSCs	Adipose Derived Mesenchymal Stem Cells
AMR	Antibody Mediated Rejection
aAMBR	Acute Antibody Mediated Rejection
ALT	Alanine Transaminase
AST	Aspartate Aminotransferase
ATG	Antithymocyte Globulin
BDNF	Brain-Derived Neurotrophic Factor
BM-MSCs	Bone Marrow Mesenchymal Stem Cells
bFGF	Basic Fibroblast Growth Factor
BTNL8	Butyrophilin-like 8
BUN	Blood Urea Nitrogen
CAR-T cells	Chimeric Antigen Receptor T cells
CAR-Tregs	Chimeric Antigen Receptor-Regulatory T cells
CARs	Chimeric Antigen Receptors
CCL22	Chemokine (C-C motif) ligand 22
CCL5	C-C Motif Chemokine Ligand 5
CCL8	C-C Motif Chemokine Ligand 8
CCR7	C-C Motif Chemokine Receptor 7
CD	Cluster of Differentiation
CD45RA	Cluster of differentiation 45RA
CD86	Cluster of Differentiation 86
CICN-3	Not defined in the text (assumed abbreviation)
CMV	Cytomegalovirus
CRS	Cytokine Release Syndrome;CsA, Cyclosporine A
CSF	Colony Stimulating Factor
CTLA4-Ig	Cytotoxic T-Lymphocyte Associated Protein 4- Immunoglobulin
CXCL12	C-X-C Motif Chemokine Ligand 12
DC-SIGN	Dendritic Cell C-type Lectin Receptor
DCregs	Dendritic Cell Regulators
dsCAR Tregs	Donor-specific Chimeric Antigen Receptor Tregs
DLBCL	Diffuse Large B cell Lymphomas
DSAs	Donor Specific Antibodies
EM	Effector/Memory
eMDSC	Early-stage Myeloid-derived Suppressor Cells
FcγRs	Fc-gamma Receptors
Foxp3	Forkhead box P3
G-MDSCs	Granulocyte-like Myeloid-derived Suppressor Cells
HGF	Hepatocyte Growth Factor
HLA-A2	Human Leukocyte Antigen A2
HLA-DR	Human Leukocyte Antigen-DR
ICANS	Immune Effector Cell-associated Neurotoxicity Syndrome
IDO	Indoleamine 2,3-dioxygenase
IFN	Interferons
IFN-y	Interferon gamma
IL	Interleukin
IL-17A	Interleukin 17A
IL-6	Interleukin 6
iNOS	inducible Nitric Oxide Synthase
iPSCs	Induced Pluripotent Stem Cells
iTregs	Induced Tregs
KGF	Keratinocyte Growth Factor
mAB	Monoclonal Antibody
M-CSF	Macrophage Colony Stimulating Factor
MDSCs	Myeloid-derived Suppressor Cells
MHC	Major Histocompatibility Complex
MHCII	Major Histocompatibility Complex Class II
M-MDSCs	Monocytic Myeloid-derived Suppressor Cells
MMF	Mycophenolate mofetil
mMDSC	Monocytic Myeloid-derived Suppressor Cells
MSCs	Mesenchymal Stem Cells
NF-κB	Nuclear Factor Kappa B
NK	Natural Killer
NGF	Nerve Growth Factor
NO	Nitric Oxide
ODNs	Oligodeoxynucleotides
OPTN	Organ Procurement and Transplantation Network
PBMC	Peripheral Blood Mononuclear Cells
PD-L1	Programmed Death-Ligand 1
PGE2	Prostaglandin E2
pmnMDSC	Polymorphonuclear Myeloid-derived Suppressor Cells
pTregs	Peripheral Tregs
RAI	Rejection Activity Index
RMCs	Regulatory Myeloid Cells
SOTs	Solid Organ Transplants
TBIL	Total Bilirubin
TEFF	Effector T cells
TGF-β	Transforming Growth Factor beta
TGF-β1	Transforming Growth Factor beta 1
Th17	T helper 17 cells
TIGIT	T cell immunoreceptor with Ig and ITIM domains
TIMP-1	Tissue Inhibitor of Metalloproteinases 1
TIMP-2	Tissue Inhibitor of Metalloproteinases 2
TNF-α	Tumor Necrosis Factor alpha
TRACT	Treg Adoptive Cell Therapy
Tregs	Regulatory T cells
nTregs	Natural or thymus Tregs
UC-MSCs	Umbilical Cord Mesenchymal Stromal Cells
UNOS	United Network for Organ Sharing
VCA	Vascularized Composite Allografts
VEGF	Vascular Endothelial Growth Factor.
